# Unveiling BaTiO_3_-SrTiO_3_ as Anodes for Highly Efficient and Stable Lithium-Ion Batteries

**DOI:** 10.3390/nano14211723

**Published:** 2024-10-29

**Authors:** Nischal Oli, Nawraj Sapkota, Brad R. Weiner, Gerardo Morell, Ram S. Katiyar

**Affiliations:** 1 Department of Physics, University of Puerto Rico-Rio Piedras Campus, San Juan, PR 00925, USA; 2 Department of Physics and Astronomy, Clemson University, Clemson, SC 29634, USA; 3 Department of Chemistry, University of Puerto Rico-Rio Piedras Campus, San Juan, PR 00925, USA

**Keywords:** BaTiO_3_, SrTiO_3_, perovskite-type materials, high-rate performance, lithium-ion batteries

## Abstract

Amidst the swift expansion of the electric vehicle industry, the imperative for alternative battery technologies that balance economic feasibility with sustainability has reached unprecedented importance. Herein, we utilized Perovskite-based oxide compounds barium titanate (BaTiO_3_) and strontium titanate (SrTiO_3_) nanoparticles as anode materials for lithium-ion batteries from straightforward and standard carbonate-based electrolyte with 10% fluoroethylene carbonate (FEC) additive [1M LiPF_6_ (1:1 EC: DEC) + 10% FEC]. SrTiO_3_ and BaTiO_3_ electrodes can deliver a high specific capacity of 80 mA h g^−1^ at a safe and low average working potential of ≈0.6 V vs. Li/Li^+^ with excellent high-rate performance with specific capacity of ~90 mA h g^−1^ at low current density of 20 mA g^−1^ and specific capacity of ~80 mA h g^−1^ for over 500 cycles at high current density of 100 mA g^−1^. Our findings pave the way for the direct utilization of perovskite-type materials as anode materials in Li-ion batteries due to their promising potential for Li^+^ ion storage. This investigation addresses the escalating market demands in a sustainable manner and opens avenues for the investigation of diverse perovskite oxides as advanced anodes for next-generation metal-ion batteries.

## 1. Introduction

A gift to modern humanity, lithium-ion batteries (LIBs) embodied by high energy and power density, enhanced safety features, and prolonged life cycles are presently propelling advancements in the domain of portable electronics, automobiles, electric vehicles (EVs) and hybrid electric vehicles (HEVs) [1,2,3,4]. Exploring novel stable materials for negative electrodes is essential for improving the performance of LIBs [5,6,7,8]. This is accomplished through the utilization of lithium-alloy materials such as tin, germanium, silicon, phosphorus, and lithium metal [9,10,11,12,13]. Regrettably, lithium-alloying reactions exhibit substantially large volume expansion of ~300%, resulting in significant capacity deterioration in a few cycles. The enhancement of cycle stability in rechargeable lithium-ion batteries is pursued through the design and implementation of advanced composite materials [14]. Pointedly, composite configurations such as silicon–graphite/carbon hybrids [15], red phosphorus–carbon [16], black phosphorus–graphite [17], and tin–graphene [18] are researched as effective avenues for improvement. In contrast to lithium-alloy anode materials, lithium-intercalation anode materials, mainly commercial graphite, exhibit minimal volume change during lithiation–delithiation [19]. However, graphite encounters challenges arising from poor intercalation kinetics and lithium plating phenomena, particularly fast charging at high current and low temperature [20]. These issues emanate from graphite’s low lithiation potential (≈0.01 V vs. Li/Li^+^), which turn out to be safety concerns related to dendritic lithium growth [21]. Thus, the average working voltage needs to be high enough to prevent Li plating but still low enough to accomplish a high full cell output potential. Titanium-based anode material Li_4_Ti_5_O_12_ [22,23] and niobium-based anode materials (W_5_Nb_16_O_55_, Cu_2_Nb_34_O_87_, Nb_2_O_5_, etc.) exhibit high-rate performance and extended cycle stability [24,25]. However, these materials face challenges such as low output voltage and poor electronic conductivity. The synthesis of intercalation-type anode materials with simultaneously high intrinsic electronic and ionic conductivity while preserving essential properties poses a significant hurdle in advancing the utilization of anodes in lithium-ion batteries.

In light of these challenges, it is imperative to explore advanced novel materials. One promising class of compounds is perovskites with the ABO₃ structure, characterized by large A-site cations positioned at the central lattice, which play a crucial role in stabilizing the overall framework. Its unique corner-sharing BO_6_ structure creates three-dimensional (3D) networks facilitating rapid Li^+^ transport and ensuring high ionic conductivity [26]. The perovskite family of anode materials presents a promising avenue for enhancing lithium-ion battery (LIB) performance, with specific emphasis on compounds such as La_0.5_Li_0.5_TiO_3_ [27], NdMnxFe_1−x_O_3_ [28], SrVO_3_ [29], BiFeO_3_ [5], CaMnO_3_ [30], RMnO_3_ (where R represents La, Nd, or Eu) [31], and LiNbO_3_ where the amorphous LiNbO3 films demonstrate impressive gravimetric capacity during rapid cycling. Notably, these films were cycled without the need for any conductive additives [32], indicating that they possess excellent intrinsic electronic conductivity and high Li^+^ ion diffusivity, attributed to their amorphous structure [32]. These materials exhibit significant electrochemical properties conducive to lithium storage and cycling stability. Noteworthy candidates also include Na_0.5_Bi_0.5_TiO_3_ [33] and hollow spherical LaFeO_3_ [34], alongside CeMnO_3_ nanofibers [35] and double perovskite configurations like La_2_MnNiO_6_ [36]. Hybrid perovskite-like io-dobismuthates as low-cost and stable anode materials [37], highly stable metal halide perovskite microcube anodes for lithium-air batteries [38], perovskite oxides with Pb at B-site [39], photorechargeable lead-free perovskite lithium-ion batteries using hexagonal Cs_3_Bi_2_I_9_ nanosheets [40], a microstructure engineered perovskite super anode with Li-storage life of exceeding 10,000 cycles [41]. Despite their potential, the exploration of perovskite materials as battery anode has been notably limited. SrTiO_3_ as an anode only studied by Derek C. Johnson and Amy L. Prieto in LIBs revealed a specific capacity below 100 mA h g^−1^ at a working potential of ~1 V vs. Li/Li^+^ and with lithiation potentials 0.105 V and 0.070 V vs. Li/Li^+^ and 0.095 Vand 0.142 V vs. Li/Li^+^ during delithiation [42]. The cyclability was reported to be less than 35 cycles, and capacity faded rapidly. But, BaTiO_3_ has not been researched yet as an anode for LIBs. Thus, exploring SrTiO_3_ and BaTiO_3_ offers opportunities to enhance the capacity and rate capability of LIBs anodes. It reveals the arena of novel compounds as substitutes for the existing graphite anode. Here, we report that the SrTiO_3_ and BaTiO_3_ electrodes exhibit a high specific capacity of 90 mA h g^−1^ at a low current density of 20 mA g^−1^, a safe and low average working potential of ~0.6 V vs. Li/Li^+^, which is higher than graphite anode (Supplementary Information Figure S5) while also demonstrating excellent high-rate performance, maintaining a capacity of ~80 mA h g^−1^ over 500 cycles at high current density of 100 mA g^−1^ by both compound. These findings highlight the potential of perovskite-type materials as promising candidates for anode applications in Li-ion batteries, given their capacity for efficient Li^+^ ion storage.

## 2. Experimental Section

### 2.1. Synthesis of Electrode Materials

The electrode materials were synthesized via a commercially feasible ball mill approach. Initially, commercially available BaTiO_3_ (Sigma Aldrich, nanopowder 99.99%, St. Louis, MO, USA) and SrTiO_3_ (Sigma Aldrich, nanopowder 99%, St. Louis, MO, USA) were utilized. These materials were then mixed in a ratio of 7:2 with carbon super P and subjected to grinding for an hour.

### 2.2. Materials Characterizations

Scanning electron microscopy—energy dispersive spectroscopy (SEM-EDS) analyses were performed utilizing JEOL, model JSM 6480 LV, Tokyo, Japan. X-ray diffraction (XRD) measurements were conducted employing a Rigaku SmartLab system, using Cu Kα radiation with a wavelength (λ) of 1.5408 Å, scanning within the range of 20 to 80 degrees at a rate of 2 degrees per minute. Raman spectra were attained using a T64000 Raman spectrometer from Instrument S.A., Jobin Yvon -Horiba (Longjumeau, France) Triple monochromator in subtractive mode equipped with a CCD detector. An Olympus microscope focused with an 80X objective in backscattering mode. The laser is Coherent Argon Innova 70C using 514.53 nm and 5 mW on the sample.. X-ray photoelectron spectroscopy (XPS) has also been performed to analyze detailed insights into the elemental composition and the chemical and electronic states of atoms. XPS data were collected using a PHI VersaProbe III with a monochromatic Al Kα X-ray source (hν = 1486.6 eV) and an Al anode powered at 25 W and 15 kV.

### 2.3. Electrochemical Analysis

In order to analyze the electrochemical performance, the slurry was prepared by mixing active materials (BaTiO_3_ and SrTiO_3_) with Super P carbon black as a conductive agent and Poly (vinylidene fluoride) (PVDF) binder in N-Methyl-2-pyrrolidone (NMP) in a weight ratio of 70:20:10, respectively. The prepared slurry was coated on 9 µm copper foil using a doctor-blade (MTI Corporation, Richmond, CA, USA). The coated electrodes were dried in a vacuum oven at 110 °C for 12 h. The battery performance of the electrodes was assessed by punching out 10 mm coupons and using them as the working electrode for CR2032 coin cells. Polypropylene Celgard was used as the separator, and lithium (Li) metal served as the counter/reference electrode. The electrolyte for the lithium-ion cell was 1 M LiPF_6_ in ethylene carbonate (EC)/dimethyl carbonate (DMC) [1:1 (*v*/*v*)] containing 10 wt.% of fluoroethylene carbonates (FEC) as an additive [1 M LiPF_6_/EC-DMC + 10% FEC]. The active materials (BaTiO_3_ and SrTiO_3_) had a loading of around 1.91–2.23 mg cm^−2^ with a thickness of 15–20 μm. Charge/discharge performance was evaluated with a Landt battery tester in the voltage window of 0.001–1.5 V, 0.001–2 V, and 0.001–2.5 V. The coin cells (CR2032) were assembled in an Ar-filled glove box (MBRAUN, Glovebox Workstations, Stratham, NH, USA) with H_2_O and O_2_ contents < 0.1 ppm. Fabricated coin cells were tested at various current densities using a Landt instruments battery tester (Hudson, NY, USA). Cyclic voltammetry was tested with an Arbin instrument at a scan rate of 0.1–1.2 mV s^−1^ range at 0.001–1.5 V.

## 3. Result and Discussions

**Figure 1:**
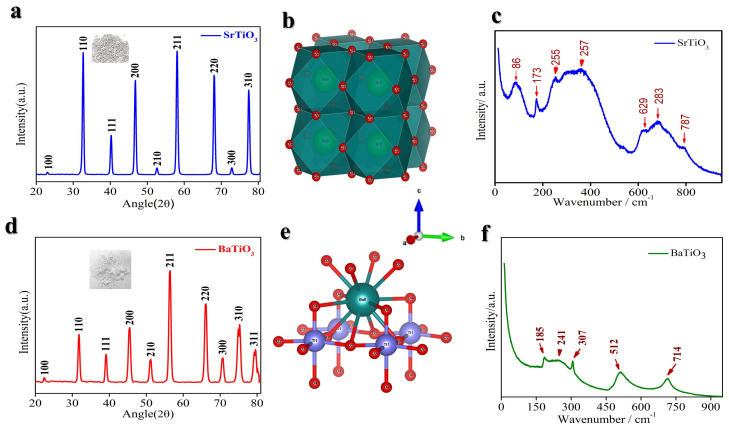
(**a**) XRD pattern SrTiO_3_ (STO). (**b**) Crystal structure of STO. (**c**) Raman shifts of STO. (**d**) XRD pattern of BaTiO_3_ (BTO). (**e**) Crystal structure of BTO. (**f**) Raman shifts of BTO.

The STO sample demonstrates itself as a fine whitish powder (Figure 1a, inset). The X-ray diffraction (XRD) pattern, depicted in Figure 1a, reveals that all observed diffraction peaks correspond to a cubic perovskite structure with space group Pm3m (#211) and a lattice constant of a = 3.90 Å, aligning with the reference value (JCPDS: 35-0734) [43]. The pronounced peaks are indicative of a high degree of crystallinity, and it has no phase impurities. The cubic crystal structure of STO is illustrated in Figure 1b, where Sr^2+^ ions are coordinated to twelve oxygen atoms, and Ti^4+^ ions are octahedrally coordinated to six oxygen atoms [44]. Figure 1c presents the Raman spectrum of the STO sample, exhibiting characteristic Raman shifts that align well with the typical Raman peaks associated with STO [45]. Moreover, the substantial mass of Sr^2+^ and Ti^4+^ ions results in a high crystal density of 5.1 g cm^−3^ [46], significantly surpassing that of graphite, thereby enhancing volumetric energy density.

Similarly, the BTO sample is a fine white powder (Figure 1d, inset). The XRD pattern indicated in Figure 1d can be readily indexed to the tetragonal BTO phase (JCPDS no. 01-075-0583) with lattice parameters a = b = 3.9950 Å and c = 4.0340 Å and crystallizes in the tetragonal P4mm (# 99) space group [47]. The well-defined peaks confirm a high degree of crystallinity and high purity in the BTO sample. BTO is a (Cubic) perovskite structure [48] shown in Figure 1e. Figure 1f illustrates the Raman spectrum of the BTO sample, displaying characteristic Raman shifts that correspond closely to the standard Raman peaks of BTO [49]. Moreover, the presence of Ba^2+^ and Ti^4+^ ions contributes to a high crystal density of 6.02 g cm^−3^ [50], which is substantially higher than that of graphite, thus enhancing volumetric energy density.

**Figure 2:**
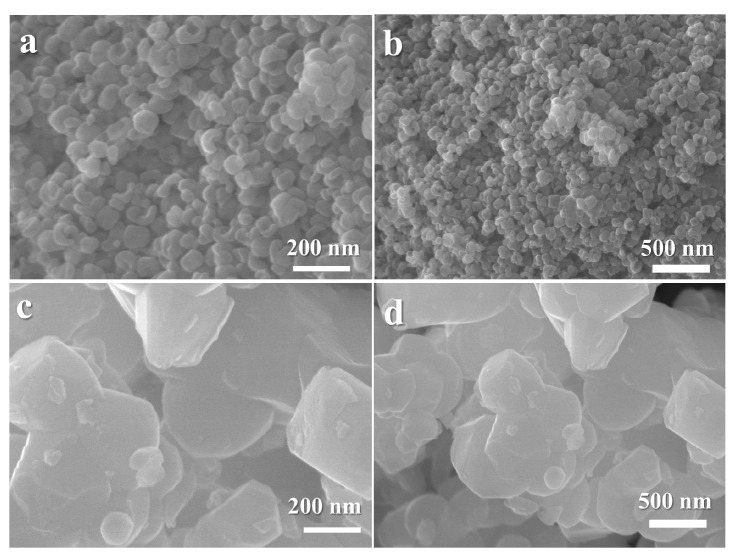
SEM images of BTO and STO. (**a**) BTO at 200 nm. (**b**) BTO at 500 nm. (**c**) STO at 200 nm. (**d**) STO at 500 nm.

Figure 2a,b illustrate the morphology of the BTO nanocrystallites. The SEM images clearly depict randomly distributed grains of smaller sizes, along with uniform spherical particles. These images reveal that the average crystalline size ranges between 20 and 100 nm.

Moreover, for STO, Figure 2c,d present SEM images, revealing particles with irregular polyhedral shapes (100–200 nm) and numerous nanosteps, consistent. The sequential layering of the particles creates a porous structure that could facilitate efficient pathways for lithium-ion migration.

**Figure 3:**
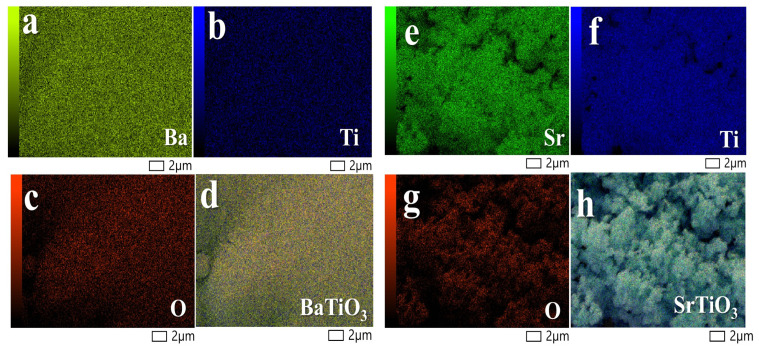
SEM mapping images of BTO and STO at 2 µm. (**a**–**d**) Ba, Ti, O, and BaTiO_3_, respectively. (**e**–**h**) Sr, Ti, O, and SrTiO_3_, respectively.

Figure 3a–d display the element mapping of BTO nanopowder, where different colors represent the distribution of Ba, Ti, and O. The EDX pattern, detailed in the (Supplementary Information Figure S1), confirms the presence of each constituent element. Both analyses indicate that BTO is uniformly distributed.

Likewise, Figure 3e–h show the elemental mapping of STO nanopowder, with distinct colors illustrating the spatial distribution of Sr, Ti, and O. The EDX pattern, provided in the (Supplementary Information Figure S2), verifies the presence of each constituent element. Both sets of data corroborate the uniform distribution of STO.

**Figure 4:**
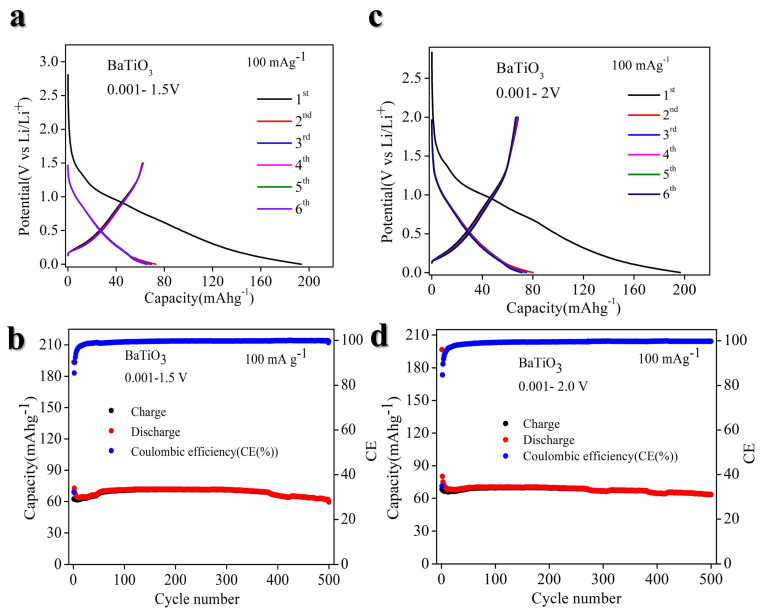
Electrochemical performance of BTO at different voltage ranges in LIBs. (**a**) Galvanostatic charge–discharge (GCD) at 0.001–1.5 V. (**b**) Cyclic performance at 0.001–1.5 V. (**c**) Galvanostatic charge–discharge (GCD) at 0.001–2.0 V. (**d**) Cyclic performance at 0.001–2.0 V.

We investigated the lithium storage capabilities of BTO (1C = 114 mA g^−1^) (in detail calculation of theoretical capacity are in Supplementary Information Page S-6) when used as an anode material for lithium-ion batteries (LIBs). The electrochemical performance of BTO was tested in three different voltage ranges: 0.001–1.5 V, 0.001–2.0 V, and 0.001–2.5 V, at room temperature. As depicted in Figure 4a, the GCD curve for the 0.001–1.5 V range shows a discharge capacity of ~200 mA h g^−1^, while the charge capacity is 70 mA h g^−1^, remaining stable over 500 cycles with ~99.99% coulombic efficiency, as shown in Figure 4b. In Figure 4c, the GCD curve for the 0.001–2.0 V range indicates a specific capacity of about 80 mA h g^−1^, which is maintained for over 500 cycles, as demonstrated in Figure 4d. Additionally, we tested the charge–discharge performance at 0.001–2.5 V, but there is no significant difference we observed, as shown in (Supplementary Information Figure S3b). However, cycling performance is poor, as demonstrated in (Supplementary Information Figure S4b).

**Figure 5:**
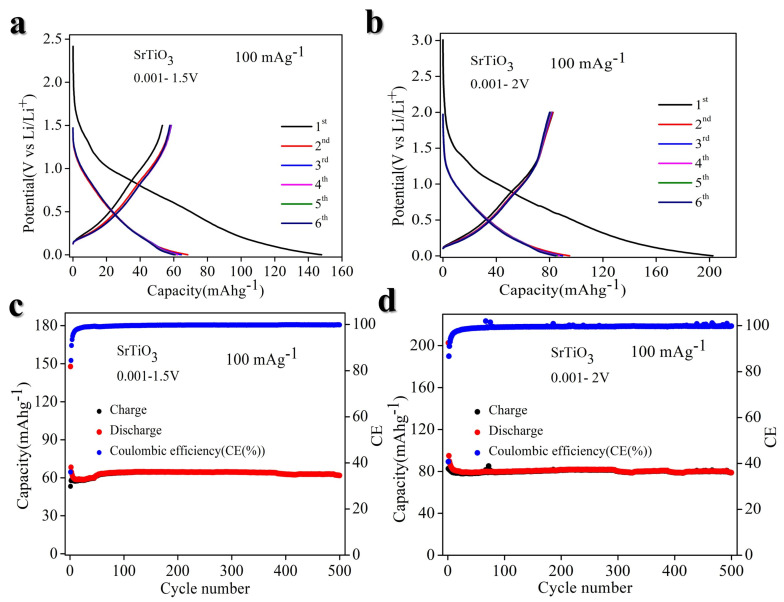
Electrochemical performance of STO at different voltage ranges in LIBs. (**a**) Galvanostatic charge–discharge (GCD) at 0.001–1.5 V. (**b**) Cyclic performance at 0.001–1.5 V. (**c**) Galvanostatic charge–discharge (GCD) at 0.001–2.0 V. (**d**) Cyclic performance at 0.001–2.0 V.

Similarly, we explored the lithium storage capabilities of STO (1C = 146 mA g^−1^) (in detail in Supplementary Information Page S-6). The electrochemical performance of STO was evaluated across three distinct voltage ranges: 0.001–1.5 V, 0.001–2.0 V, and 0.001–2.5 V, all measured at room temperature. As illustrated in Figure 5a, the GCD curve for the 0.001–1.5 V range exhibits a discharge capacity of approximately 200 mA h g^−1^, with a corresponding charge capacity of 70 mA h g^−1^, demonstrating stability over 500 cycles with ~99.99% coulombic efficiency, as shown in Figure 5b. In Figure 5c, the GCD curve for the 0.001–2.0 V range indicates a specific capacity of around 80 mA h g^−1^, which is sustained over 500 cycles, as evidenced in Figure 5d. Furthermore, the charge–discharge response at 0.001–2.5 V was examined, revealing no significant differences, as depicted in the GCD curve ((Supplementary Information Figure S3a)) but poor cycling performance, as shown in (Supplementary Information Figure S4a).

**Figure 6:**
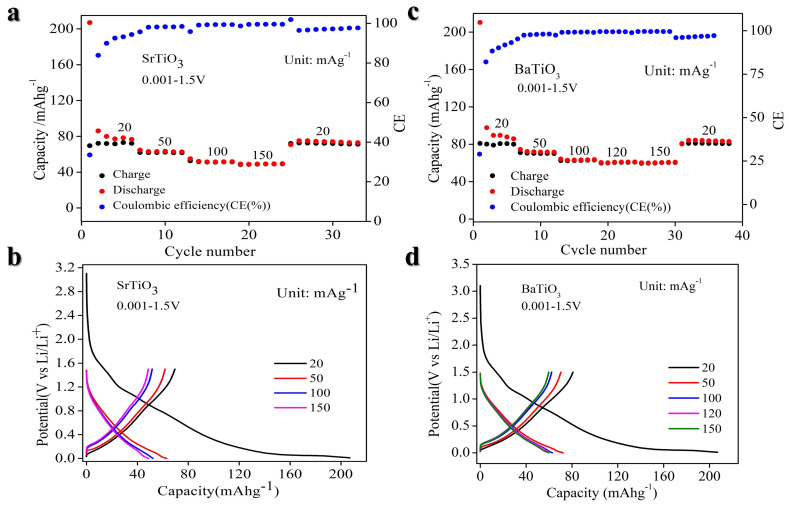
Rate performance of STO and BTO in 0.001–1.5 V. (**a**) STO rate performance in the range of 20 to 150 mA g^−1^. (**b**) STO rate curve profile at different current densities in the range of 20 to 150 mA g^−1^. (**c**) BTO rate performance in the range of 20 to 150 mA g^−1^. (**d**) BTO discharge/charge voltage profile at different current densities in the range of 20 to 150 mA g^−1^.

To understand rate capability, Figure 6a,b show the rate performance of STO and BTO at a current density ranging from 20 mA g^−1^ to 150 mA g^−1^. The data unequivocally demonstrate that both materials exhibit superior rate performance.

In regard to STO rate performance, as shown in Figure 6a,b, the discharge capacity is ~90 mA h g^−1^ at a low current density of 20 mA g^−1^, and the discharge capacity is 63 mA h g^−1^, 52 mA h g^−1^, and 50 mA h g^−1^ at the current density of 50 mA g^−1^, 100 mA g^−1^, and 150 mA g^−1^, respectively. When the current returns to 20 mA g^−1^, the discharge capacity can maintain a value of ~86 mA h g^−1^.

Similarly, for BTO, as demonstrated in Figure 6c,d, the discharge capacity is 100 mA h g^−1^ at a low current density of 20 mA g^-1^. At current densities of 50 mA g^−1^, 100 mA g^−1^, 120 mA g^−1^, and 150 mA g^−1^, the discharge capacities are 73 mA h g^−1^, 63 mA h g^−1^, 61 mA h g^−1^, and 60 mA h g^−1^, respectively. When the current returns to 20 mA g^−1^, the discharge capacity remains stable at ~100 mA h g^−1^. It reveals that both STO and BTO materials are highly reversible. The change in discharge capacity during the first cycle arises from differences in the applied potential window. The broader potential range is likely to enable additional electrochemical reactions or increased lithiation/delithiation processes, thereby influencing the overall discharge capacity.

At wider voltage ranges, the stability of the solid electrolyte interphase (SEI) becomes critical. The SEI layer can become unstable under high voltage conditions, leading to increased side reactions that consume active lithium and degrade capacity. This instability is exacerbated by the presence of impurities or defects in the anode material that can promote unwanted reactions. In this context, BTO and STO were evaluated across three voltage ranges: 0.001–1.5 V, 0.001–2.0 V, and 0.001–2.5 V. The results indicated that the 0.001–1.5 V range exhibited superior stability in performance compared to the higher voltage settings. This suggests that limiting the operational voltage can enhance the integrity of the SEI, thereby improving overall battery efficiency and longevity [51].

**Figure 7:**
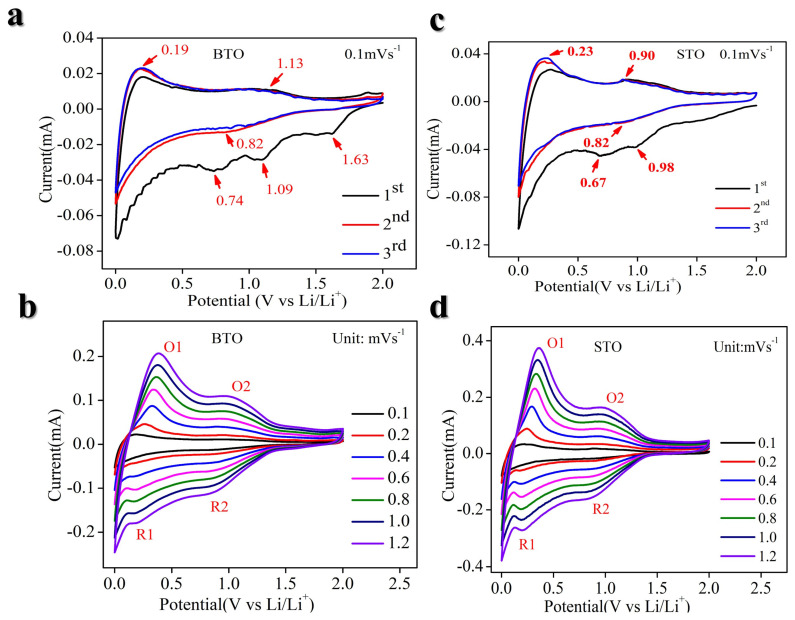
Cyclic voltammetry (CV) curves of the BTO and STO at room temperature. (**a**) CV of BTO in the first 3 cycles at a scan rate of 0.1 mV s^−1^. (**b**) CV of BTO in the different scan rates of 0.1–1.2 mV s^−1^. (**c**) CV of STO in the first 3 cycles at a scan rate of 0.1 mV s^−1^. (**d**) CV of STO in the different scan rates of 0.1–1.2 mV s^−1^.

The cyclic voltammetry profiles of BTO electrodes scanned at a rate of 0.1 mV s^−1^ reveal the presence of one broad reduction peak at 0.82 V vs. Li/Li^+^ first cycles, accompanied by two corresponding broad oxidation peaks at 1.13 and 0.19 V vs. Li/Li^+^ (Figure 7a). The galvanostatic lithiation–delithiation characterization of the BTO electrode exhibits an initial lithiation capacity of approximately 200 mA h g^−1^ and a delithiation capacity of 80 mA h g^−1^ during the first cycle, resulting in an initial Coulombic efficiency (CE) of 33% (Figure 4c). The observed low initial CE is attributable to the formation of the solid electrolyte interphase (SEI) below 1 V vs. Li/Li^+^ (Figure 4c), structural disintegration, and potential side reactions [52]. Enhancing the initial CE can be improved through the design of protective coatings or the modification of electrolyte composition by introducing suitable additives [53]. The CE increases to 85% in the second cycle and average CE ~99.9% in subsequent cycles (Figure 4a,b). A maximum delithiation capacity of ~100 mA h g^−1^ at 20 mA g^−1^ is achieved.

Similarly, for STO, the cyclic voltammetry (CV) analysis of STO electrodes at a scan rate of 0.1 mV s^−1^ demonstrates distinct electrochemical signatures. Specifically, the CV profile reveals a broad cathodic peak at approximately 0.82 V vs. Li/Li+ during the first cycle, accompanied by two corresponding broad anodic peaks at around 0.23 and 0.90 V vs. Li/Li^+^ (Figure 7c). The galvanostatic lithiation–delithiation tests indicate an initial lithiation capacity of approximately 200 mA h g^−1^ and a delithiation capacity of 80 mA h g^−1^, resulting in a low initial Coulombic efficiency (CE) of 40% (Figure 5b). This low initial CE is attributed to the formation of a solid electrolyte interphase (SEI) below 1 V vs. Li/Li^+^, structural degradation, and potential side reactions. Strategies to enhance the initial CE include the application of protective coatings or modifications to the electrolyte composition by incorporating suitable additives. Notably, the CE improves to 94% in the second cycle and stabilizes at an average CE of approximately 99.9% in subsequent cycles (Figure 5a,b). The electrode achieves a maximum delithiation capacity of approximately 90 mA h g^−1^ at a current density of 20 mA g^−1^. The galvanostatic lithiation–delithiation profiles of STO and BTO electrodes at various specific currents exhibit sloping profiles without distinct potential plateaus, indicating a solid-solution mechanism [27,54] (Figure 4a,b and Figure 5a,b). Additionally, at a current density of 20 mA g^−1^, both STO and BTO electrodes demonstrate a low and stable average working potential of ~0.6 V vs. Li/Li^+^. Compared to previously reported anode materials such as spinel Li_4_Ti_5_O_12_ [55] and perovskite Li_0.5_La_0.5_TiO_3_ [27], the perovskite STO and BTO electrodes exhibit a lower average working potential (Figure 4a,b and Figure 5a,b), underscoring the potential of STO and BTO as promising anode materials for Li-ion batteries. The capacity can be increased by doping suitable elements.

Cyclic voltammetry (CV) works as an efficient technique to study the kinetics of electrode reactions as well. Theoretically, the voltametric response of an electroactive material at different scan rates can be described by the following power–law relationship [5,56], as in Equations (1) and (2):

*i* = av^b^ (1)

log *i*(v) = b log v + log a (2)

**Figure 8:**
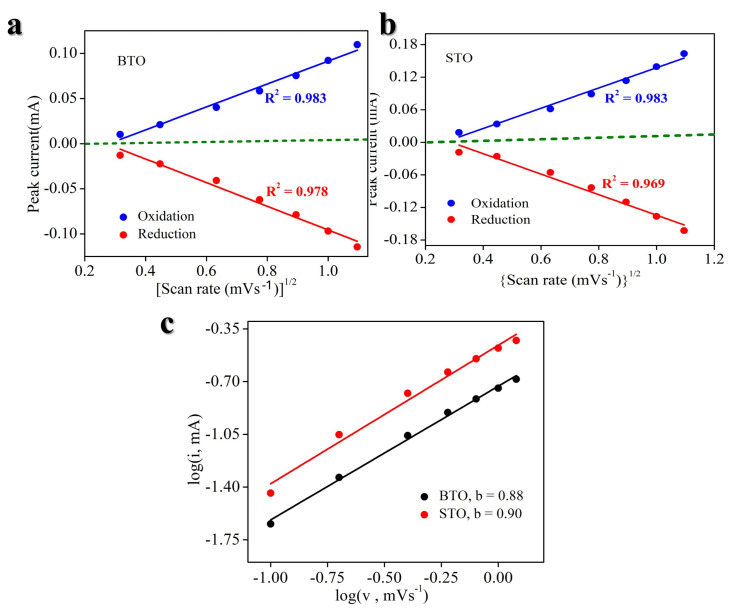
(**a**) Peak current (i) vs. v^1/2^ (square root of scan rate (v)) of BTO. (**b**) Log (i) (peak current (i)) vs. log (v) (scan rate (v)) of STO. (**c**) Log (i) vs. log (v) of BTO and STO.

Here, the response current (*i*) determined under a certain potential (V) follows a power–law dependence on the potential scan rate (v). Where the b-value, which is determined by the ion storage mechanism, can be calculated from the slope of the log (*i*)−log (v) plot. Specifically, a b-value of 0.5 indicates a completely diffusion-controlled process, while a b-value of 1.0 signifies either a faradaic contribution from charge transfer with surface or subsurface atoms (pseudocapacitance effect) or a non-faradaic contribution from the electrical double-layer effect [54]. It has been well established that across a broad range of scan rates v, conventional materials LiCoO_2_, LiFePO_4,_ and graphite demonstrate b ≈ 0.5. In contrast, pseudocapacitor materials, for example, Nb_2_O_5_, RuO_x,_ and MnO_2_ materials, exhibit b ~ 1.0. This b-value should be a crucial reason for the much higher power capabilities of supercapacitors when compared to traditional rechargeable batteries (such as Li-ion, Ni-Cd, Pb-acid, and Ni-MH batteries) [54]. As shown in Figure 8a,b, the fact that the adjusted R^2^ values anodic and cathodic of the linear fitting are approximately 1 is significant, as it indicates excellent chemical reversibility of the BTO and STO materials during charge and discharge processes, even at high scan rates [54]. As illustrated in Figure 8c, the redox reactions (i.e., charge/discharge processes) of the BTO- and STO-based electrodes exhibit b values of 0.88 and 0.90, indicating fast capacitive-controlled kinetics [27,54]. The data presented in Figure 8c further demonstrate that the electrode kinetics of the BTO- and STO-based electrodes are not controlled by diffusion processes only.

**Figure 9:**
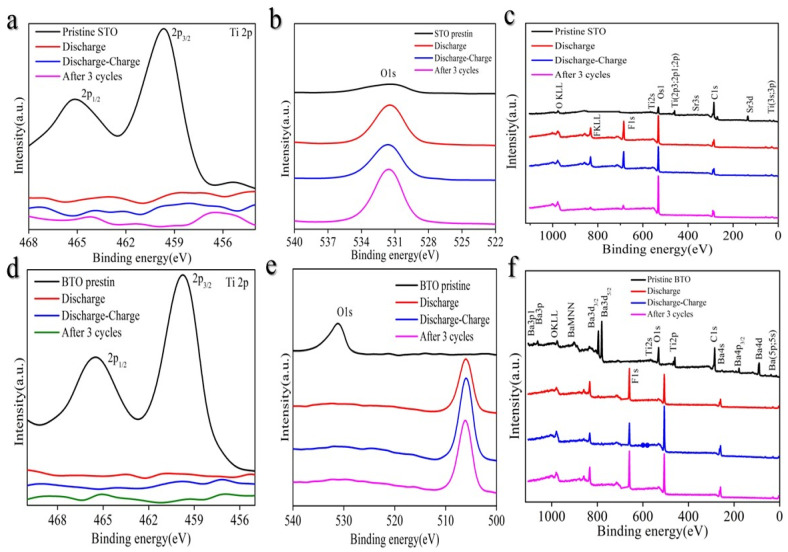
X-ray photoelectron spectroscopy (XPS) measurement. (**a**) Pristine STO, lithiation (discharge), lithiation–delithiation (discharge–charge), after 3 cycles. (**b**) O1s. (**c**) STO survey different conditions. (**d**) Pristine BTO, lithiation (discharge), lithiation–delithiation (discharge–charge), after 3 cycles. (**e**) O1s. (**f**) BTO survey different conditions.

In order to understand the Li^+^ storage mechanism STO (Figure 9a–c), X-Ray photoelectron spectroscopy (XPS) is utilized to explore the surface valence evolution to gain further insights into the origin capacity. The energy difference between O1s and Ti 2p_3/2_ levels ΔE increased to a higher value during the lithiation process (Figure 9a), evidencing the decrease in the Ti valence state [27]. At the same time, the Sr3d peak shows no obvious shift as expected for electrochemically inert Sr [29]. After full lithiation down to ≈0.001 V vs. Li/Li^+^, no obvious Ti2p (Figure 9a) and Sr3d (Supporting Information Figure S6) peaks can be seen in the XPS spectrum. After delithiation to ≈2 V vs. Li/Li^+^, the intensities of Ti 2p (Figure 9a) and Sr 3d (Supplementary Information Figure S6) peaks increase back. Also, after three cycles, Ti2p and Sr3d are also increased as before (Supplementary Information Figure S6).

In a similar manner, to elucidate the Li^+^ storage mechanism in BTO (Figure 9d–f), XPS is employed to investigate the evolution of surface valence states, thereby gaining deeper insights into the origin of capacity. During the lithiation process, the energy difference (ΔE) between the O1s and Ti 2p_3/2_ levels increases, indicating a reduction in the Ti valence state as in Figure 9d. Simultaneously, the Ba3d peak remains largely unchanged (Supplementary Information Figure S6), consistent with the electrochemically inert nature of Ba as Sr. Upon full lithiation down to approximately 0.001 V vs. Li/Li^+^, the XPS spectrum reveals the absence of evident Ti 2p (Figure 9d). Upon delithiation to around 2 V vs. Li/Li^+^, the intensities of the Ti 2p peaks increase back.

We confirm that the XPS measurements were conducted ex situ. It can be pointed out that the surface of the cycled electrodes could have been influenced by the ex situ procedure, potentially leading to contamination or modifications during the transfer process. Ex situ methods can alter the electrode surface due to exposure to air or moisture after removal from the electrochemical cell, potentially leading to contamination or surface modifications. As a result, the changes detected by XPS may not fully represent the surface state during electrochemical cycling but rather reflect artifacts introduced during handling. Although XPS is a powerful tool for surface analysis, these limitations of ex situ techniques should be considered. In contrast, in situ or operando approaches offer more accurate analysis by preventing atmospheric exposure during measurement.

**Figure 10:**
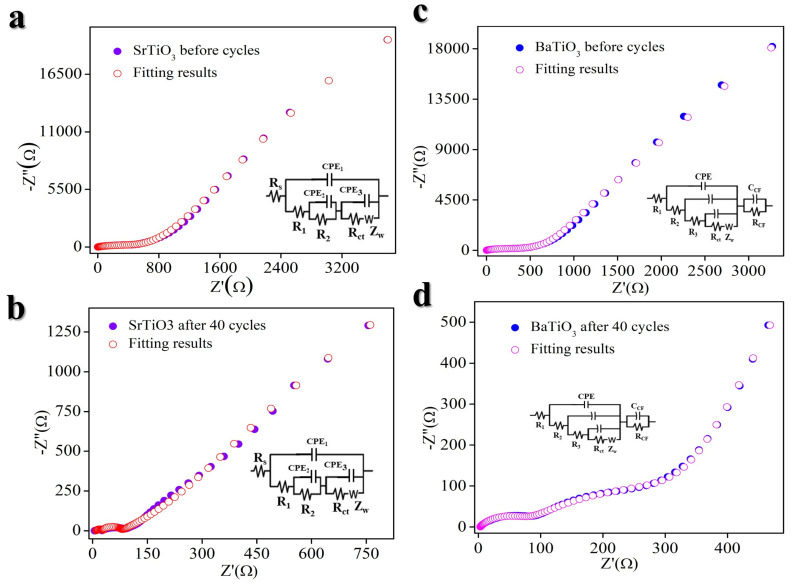
Electrochemical impedance spectroscopy (EIS) STO- and BTO-based electrodes. (**a**) STO before charging. (**b**) STO after 40 charge–discharge. (**c**) BTO before charging. (**d**) BTO after 40 cycles.

Figure 10a–d present the electrochemical impedance spectroscopy (EIS) [57] data for STO- (Figure 10a,b) and BTO (Figure 10c,d)-based electrodes under various conditions, specifically prior to charging and following 40 cycles. The EIS spectra can be divided into three primary electrical components: internal resistance (R_Ω_), electric double-layer capacitance (C_d_), and faradaic impedance (Z_f_). Faradaic impedance (Z_f_) is further subdivided into charge transfer and mass transfer components, corresponding to high- and low-frequency regions, respectively [57,58]. The charge transfer component can be represented by charge transfer resistance (R_ct_) and Warburg impedance (Z_w_). The initial cell before charging displays low charge transfer resistance (R_ct_). After 40 cycles, the R_ct_ progressively decreases, indicating the excellent stability and performance of the BTO- and STO-based electrodes.

Faradaic impedance (Z_f_) is further subdivided into charge transfer and mass transfer components, corresponding to high- and low-frequency regions, respectively. The charge transfer component can be represented by charge transfer resistance (R_ct_) and Warburg impedance (Z_w_). The initial cell before charging displays low charge transfer resistance (R_ct_). After 40 cycles, the R_ct_ progressively decreases, indicating the excellent stability and performance of the BTO- and STO-based electrodes. It is that different circuit models can potentially provide equally good fits for the measured EIS (Electrochemical Impedance Spectroscopy) data. However, while various EECs may achieve a good fit for the Nyquist plot, it is essential that each element in the selected circuit has a clear physical meaning and accurately reflects the underlying electrochemical processes. Proper assignment and interpretation of these elements are crucial to ensure that the model represents the system’s true behavior rather than merely offering a good mathematical fit.

## 4. Conclusions

In this investigation, we examined the Perovskite-based oxide compounds, namely BaTiO_3_ and SrTiO_3_ nanoparticles, as anode materials for lithium-ion batteries utilizing commercial nanopowders, SrTiO_3_ and BaTiO_3_ with standard carbonate-based electrolyte composed of 1M LiPF_6_ (1:1 EC:DEC) with 10% FEC. The SrTiO_3_ and BaTiO_3_ electrodes exhibited a remarkable specific capacity of ~90 mA h g^−1^ at a low current density of 20 mA g^−1^, a stable and low average operating potential of approximately 0.6 V vs. Li/Li^+^, in addition to outstanding high-rate performance, maintaining a capacity of around 80 mA h g^−1^ over an extended cycling period of approximately 500 cycles at a high current density of 100 mA g^−1^. Our findings demonstrate the feasibility of directly employing perovskite-type materials as anodes in lithium-ion batteries, underscoring their significant potential for lithium-ion storage. This research addresses the increasing market demands sustainably and paves the way for further exploration of various perovskite oxides as advanced anode materials for next-generation metal-ion batteries.

## Data Availability

Data can be available upon request.

## Supplementary Materials

The following supporting information can be downloaded at: https://www.mdpi.com/article/10.3390/nano14211723/s1, Figure S1: BTO energy dispersive X-Ray (EDX) spectrum. Figure S2: STO energy dispersive X-Ray (EDX) spectrum. Figure S3: (a) STO GCD profile at 0.001–2.5 V. (b) BTO GCD profile at 0.001–2.5 V. Figure S4: (a) STO cyclic performance at 0.001–2.5 V. (b) BTO cyclic performance at 0.001–2.5 V. Figure S5: Graphite, BTO, and STO GCD profile at 100 mA g^−1^. Page S-6: The theoretical capacity of BaTiO_3_ and SrTi O_3_ electrode calculation. Figure S6: XPS of BTO (a) Before charging. (b) Discharge (lithiation). (c) Discharge–charge (lithiation–delithiation). (d) After 3 cycles. Figure S7: XPS of STO. Before charging, discharge (lithiation), discharge–charge (lithiation–delithiation), after 3 cycles.
