# Supplementary material for: Unveiling BaTiO3-SrTiO3 as Anodes for Highly Efficient and Stable Lithium-Ion Batteries
Source: Nanomaterials (Basel). 2024 Oct 29;14(21):1723. doi: 10.3390/nano14211723 (PMC11547623; doi:10.3390/nano14211723)
Supplement: Supplementary file 1 [file nanomaterials-14-01723-s001.zip › nanomaterials-3238111-supplementary.pdf]

Supplementary Information

Unveiling $BaTiO_3$-$SrTiO_3$ as Anodes for Highly Efficient and Stable Lithium-Ion Batteries

Nischal Oli [1,*], Nawraj Sapkota [2], Brad R. Weiner [3], Gerardo Morell [1] and Ram S. Katiyar [1,*]

1 Department of Physics, University of Puerto Rico-Rio Piedras Campus, San Juan, PR 00925-2537, USA; gerardo.morell@upr.edu
2 Department of Physics and Astronomy, Clemson University, Clemson, SC 29634, USA; nsapkot@g.clemson.edu
3 Department of Chemistry, University of Puerto Rico-Rio Piedras Campus, San Juan, PR 00925-2537, USA; brad.weiner@upr.edu
* Correspondence: nischal.oli@upr.edu (N.O.); ram.katiyar@upr.edu (R.S.K.)

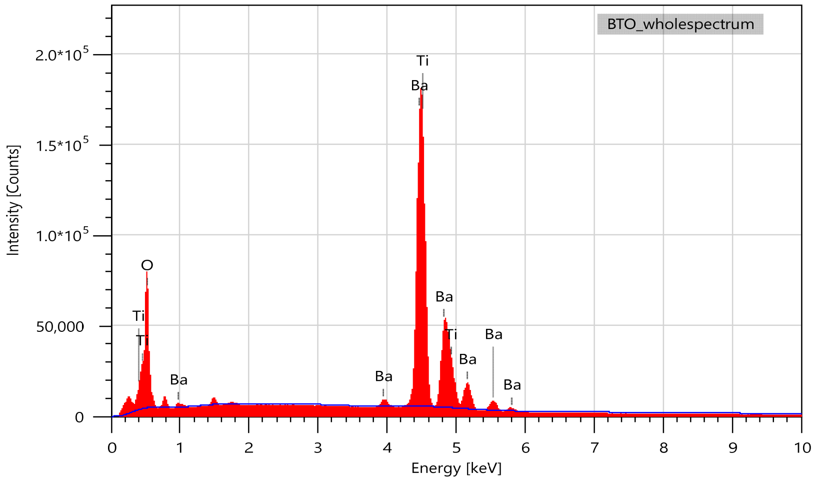

O	K	17.39±0.03	54.78±0.08
Ti	K	21.73±0.03	22.87±0.03
Ba	L	60.89±0.05	22.35±0.02
Total		100.00	100.00

Figure S1. BTO Energy dispersive X-ray (EDX) spectrum.

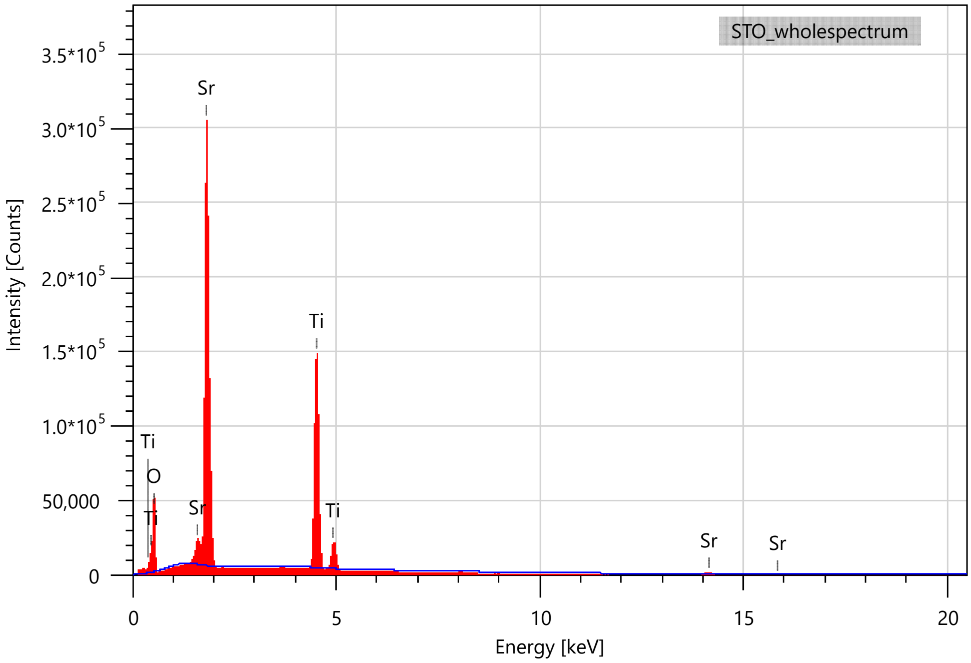

O	K	22.91±0.04	55.29±0.10
Ti	K	29.39±0.02	23.69±0.02
Sr	L	47.70±0.04	21.02±0.02
Total		100.00	100.00

Figure S2. STO Energy dispersive X-ray (EDX) spectrum.

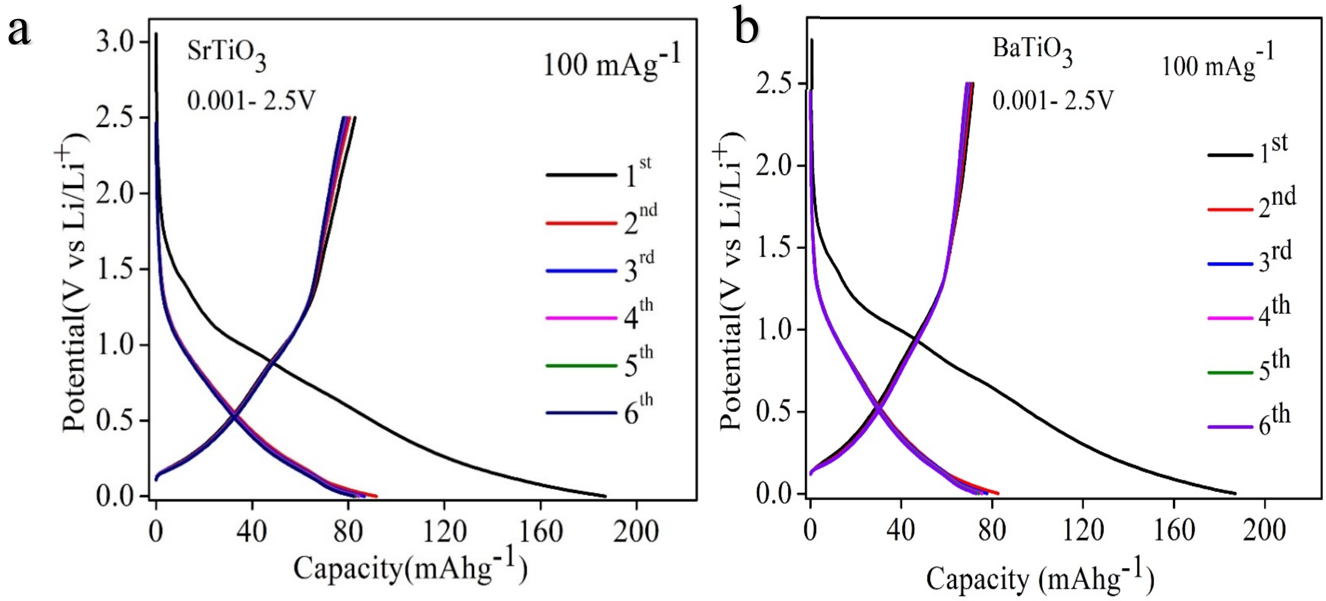

Figure S3. a) STO GCD profile at 0.001- 2.5 V. **b)** BTO GCD profile at 0.001- 2.5 V.

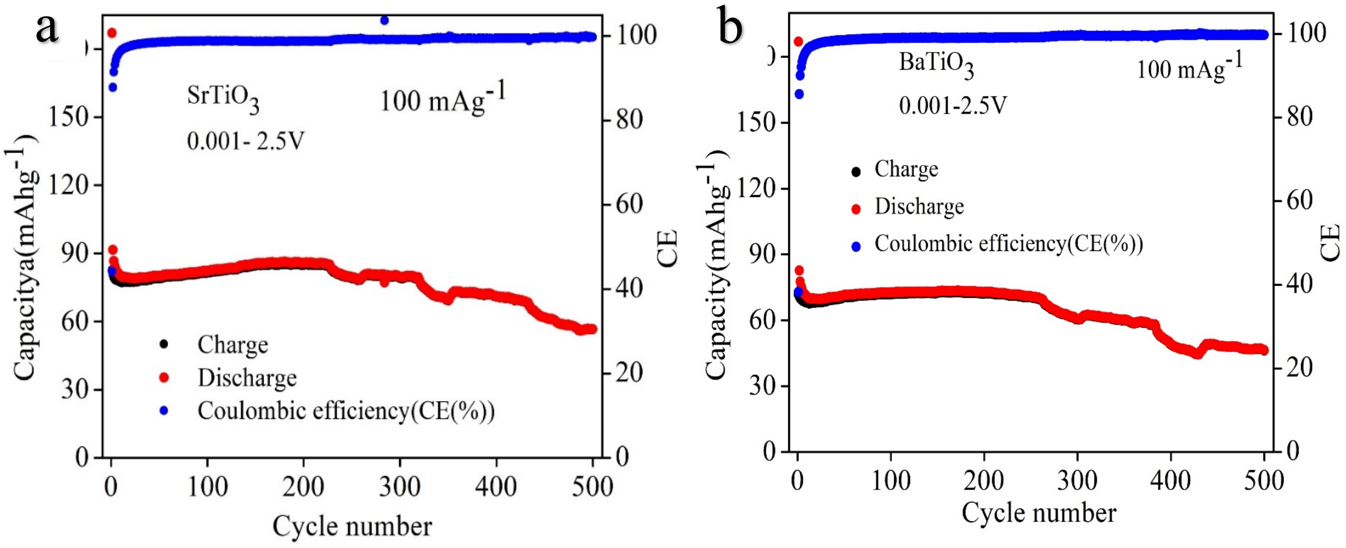

Figure S4. a) STO cyclic performance at 0.001- 2.5 V. **b)** BTO cyclic performance at 0.001- 2.5 V.

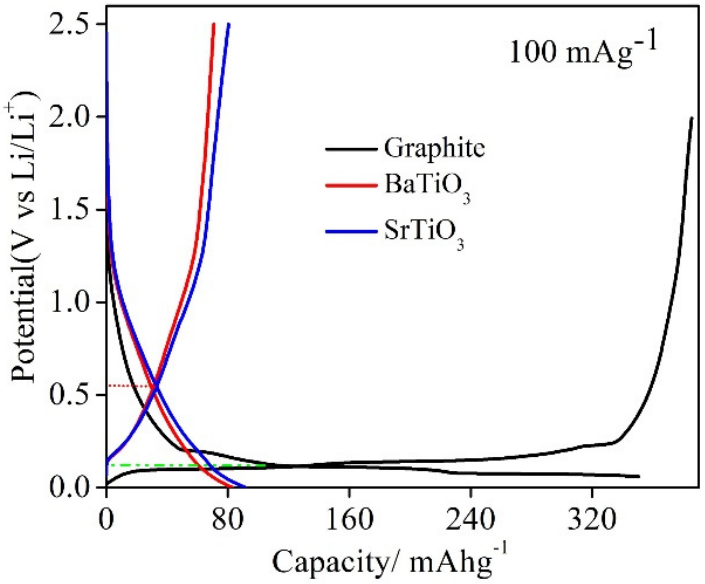

Figure S5. Graphite, BTO and STO GCD profile at 100 mA g^{-1}.

The theoretical capacity of $BaTiO_3$ and SrTi O_3 electrode calculation

The theoretical capacity (C, mA h g^{-1}) can be determined using the following equation:

$$C = \frac{n \times F}{3.6 \times M}$$

Where:

- n is the number of exchanged Li^+ ions per unit cell.
- F is the Faraday constant, 96485 C mol^{-1}
- M is the molecular weight of the material.

For the $SrTiO_3$:

- The molecular weight M is 183.49 g mol^{-1}.
- With the intercalation of 1 Li^+/V per unit cell, the theoretical capacity is calculated as:

$$C = \frac{1 \times 96485}{3.6 \times 183.49} \approx 146.07\ mA\ h\ g^{-1}$$

For the $BaTiO_3$:

- The molecular weight M is 233.192 g mol^{-1}.
- With the intercalation of 1 Li^+/ V per unit cell, the theoretical capacity is calculated as:

$$C = \frac{1 \times 96485}{3.6 \times 233.192} \approx 114.93\ mA\ h\ g^{-1}$$

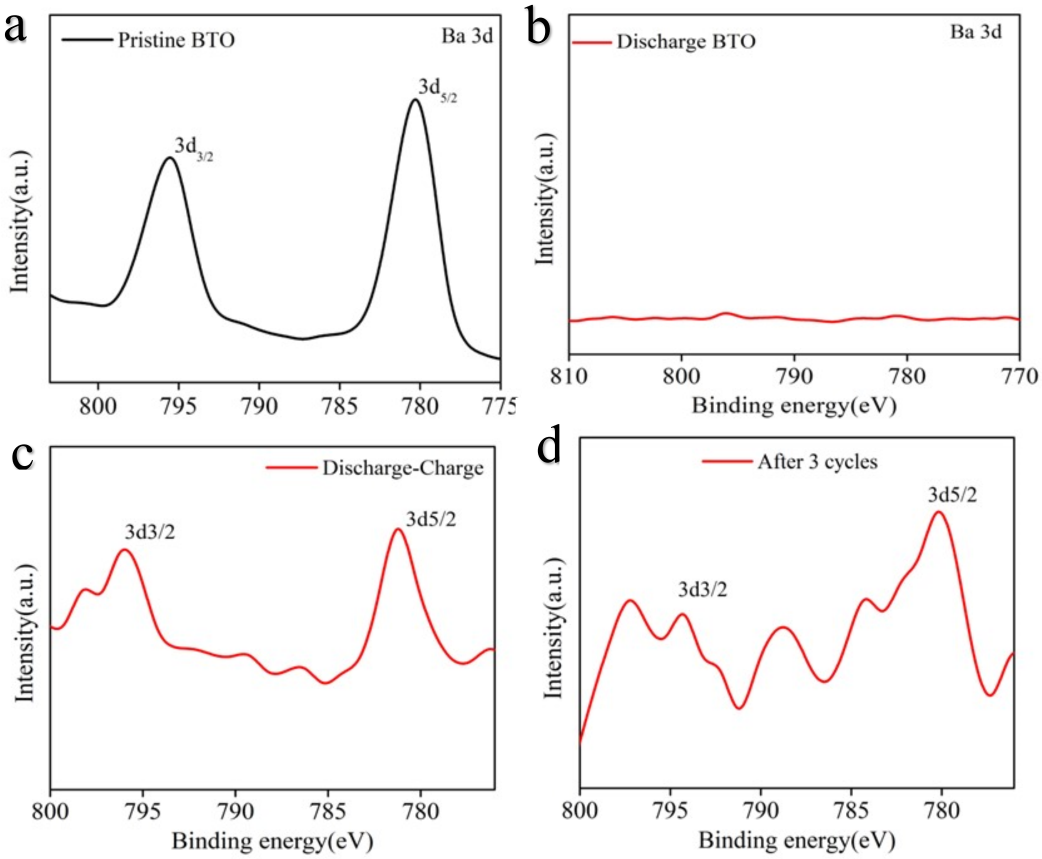

Figure S6. XPS of BTO of Ba3d a) Before charging. b) Discharge (lithiation). c) Discharge-charge (lithiation -delithiation). d) After 3 cycles.

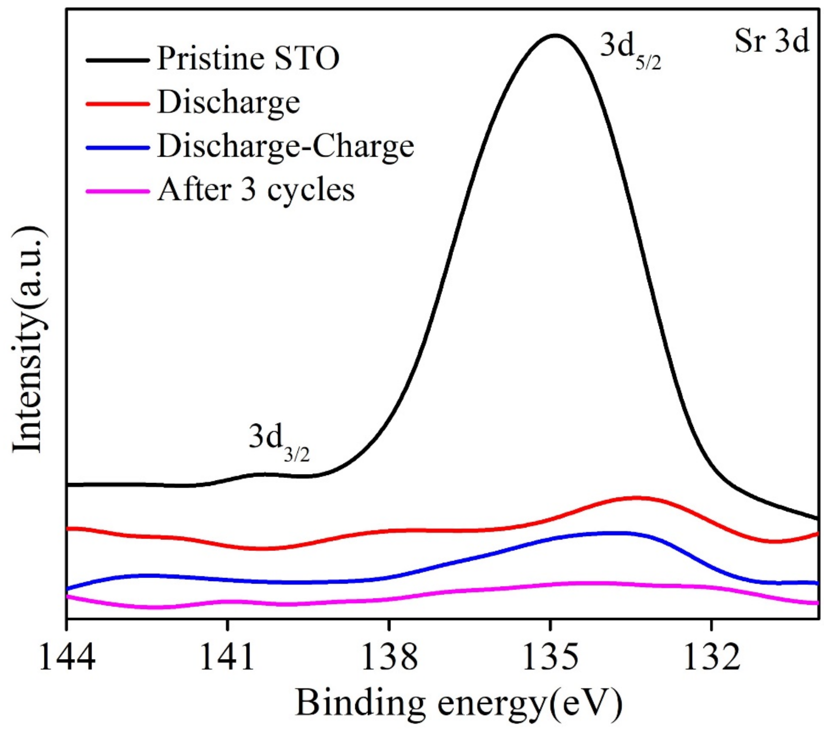

Figure S7. XPS of STO of Sr3d: Before charging, Discharge (lithiation), Discharge-charge (lithiation -delithiation), After 3 cycles.
